# Evaluating Psychometric Properties of the New Teachers’ Perceptions of Collective Efficacy to Handle Bullying Scale (TCEB)

**DOI:** 10.3390/ijerph182111424

**Published:** 2021-10-30

**Authors:** Ana Carolina Reyes-Rodríguez, Angel Alberto Valdés-Cuervo, Lizeth Guadalupe Parra-Pérez, Fernanda Inéz García-Vázquez, Gisela Margarita Torres-Acuña

**Affiliations:** Department of Education, Technological Institute of Sonora, Obregón 85000, Mexico; reyesanacarolina@gmail.com (A.C.R.-R.); lizparra@gmail.com (L.G.P.-P.); fernanda.garcia@itson.edu.mx (F.I.G.-V.); gisela.torres@itson.edu.mx (G.M.T.-A.)

**Keywords:** collective efficacy, bullying, teachers, internal structure, external validity, measurement invariance

## Abstract

Collective efficacy is a promising theoretical construct that has been used to explain bullying rates in school. The development of school collective efficacy scales has increased in bullying research in recent years; however, gaps remain in measuring collective efficacy to handle bullying. This research assessed the psychometric properties of a new scale to evaluate collective efficacy against bullying. This first-order one-dimensional scale is called the teachers’ perceptions of collective efficacy to handle bullying (TCEB) scale. A sample of 804 Mexican primary teachers completed questionnaires. The sample was randomly split into two subsamples for calibration (*n* = 402) and cross-validation analysis (*n* = 402). The factor structure was supported by confirmatory factorial analysis. Measurement equivalence was confirmed by gender. The latent means differences showed no statistically significant differences by teachers’ gender. The TCEB correlation with school environment factors (e.g., principal support, school climate, and bullying) confirms the scale’s discriminant and concurrent validity. Our findings suggest that TCEB is a suitable instrument to assess teachers’ perceptions of collective efficacy to handle bullying, a construct that has proved to help predict a positive whole-school context and student bullying involvement.

## 1. Introduction

Bullying is a pervasive worldwide school problem [1,2], with disturbing effects on students (e.g., well-being, psychosocial adjustment, and school engagement) [3,4,5]. Bullying involves a repeated and intentional aggression from students with some physical or social advantage over peers with difficulty defending themselves [6]. Many scholars in the field [7,8] consider the social-ecological model [9] to be an accurate framework in studying bullying due to its scope in exploring the phenomenon and the social context component.

Scholars also highlight the role of a school’s culture and relationships in regulating aggressive behavior [10,11]. Empirical research confirms that whole-school variables have a considerable effect on bullying rates [10,11]. Some studies [12,13,14,15] underline teachers’ effective interventions can consistently reduce bullying behaviors in students. Nonetheless, teachers do not work isolated; they belong to school communities that clearly influence the way they cope with bullying incidents [16,17].

### 1.1. Teachers’ Perception of Collective Efficacy

Research on human agency has underlined the positive effects of personal efficacy in cognitions, feelings, and behaviors. However, the literature also acknowledges an interdependence between human behavior, social and institutional environment. In other words, personal efficacy development depends not only on individual assets but also on social and institutional resources with which individuals enter into contact. Given its relevance, the Social Cognitive Theory (SCT) [18,19] has extended the concept of personal efficacy to collective efficacy [16,20]. Collective efficacy alludes to individual beliefs about a group’s capability to work together and achieve commons goals [21,22,23,24]. Similar to self-efficacy, the beliefs of members of a group about collective efficacy influence their effort and perseverance to achieve common goals [18,19].

In education, teachers’ perceptions of collective efficacy refer to a personal judgment about their fellow staff members’ capabilities to execute educational practices that support educational and psychosocial adjustment in school [25]. Collective efficacy has attracted the attention of educational research due to its positive effects on the two main education stakeholders: teachers and students. Specifically, some studies [25,26,27] underline an association between teachers’ perceptions of collective efficacy and their satisfaction, professional commitment, and support for student achievement. Also, evidence [28,29,30] shows that teachers’ collective efficacy positively affects students’ self-efficacy, school engagement, interpersonal behaviors, and higher levels of academic achievement. However, the positive effects of teachers’ collective efficacy are not limited to individual wellness; it seems to influence positive behavior in the entire school community. In specific, in bullying research, some studies found a negative association between teacher perception of collective efficacy in handling bullying and bullying rates [31,32,33]. A recent systematic review confirms that teachers’ perception of collective efficacy is critical in explaining differences in bullying rates [34]. In this regard, some scholars argue that teachers’ collective efficacy prevents bullying because it encourages defender teachers and defender students to intervene to stop bullying events [35,36]. Thus, the effects of collective efficacy on bullying prevention deserve to be in the spotlight in future research.

### 1.2. Measures of Collective Teachers’ Efficacy to Handle Bullying

As suggested above, the positive effects of teacher collective efficacy on bullying rates have increased the interest in measuring this construct. Although several studies [35,36,37,38,39,40] have tested this association, they are limited because they only assess students’ perspectives, despite the critical role of teachers as primary stakeholders. The assessment of collective efficacy from teachers’ perspectives should be seen as crucial since teachers’ perceptions of their fellow teaching staff’s ability to handle bullying would be reflected in their own actions and performance in such situations [21,24,41,42]. Despite its relevance, no scale known by the authors assesses collective efficacy to handle bullying from the teachers’ perspectives.

Additionally, most collective efficacy scales to handle bullying have some theoretical inconsistencies with Bandura’s theory. For instance, previous scales [34,40,43] measure abilities, locus of control, and willingness to intervene, instead of collective efficacy. Furthermore, some scholars [35,38], based on the Social Disorganization Theory [44,45] assess collective efficacy through the dimensions of social cohesion and social control (willingness to intervene for the shared goals). Although these dimensions could be essential elements for developing collective efficacy, they cannot evaluate teachers’ perceptions of teaching staff capabilities. To our knowledge, only the Barchia and Bussey [36] scale is grounded in SCT theory. Although the authors report instrument validity, measurement invariance by gender, and reliability, the scale measures students’ perception of school collective efficacy instead of teachers’ collective efficacy (e.g., How well can the students and teachers at your school work together to stop bullying).

### 1.3. Measurement Invariance

Previous studies on collective teacher efficacy to handle bullying also did not look at potential differences by gender. Additionally, any findings of differences by gender of collective teacher efficacy did not focus on bullying and were inconsistent. Some researchers found evidence that female teachers perceive higher collective efficacy than males [46,47,48], while other studies [49,50,51] found no gender differences. However, findings should be taken with caution since many studies did not test the invariance of the construct when comparing groups. The only study [52] that tested gender invariance did not find differences between groups. In line with Putnick and Bornstein [53], we believe it remains essential to verifying the measurement invariance (at least by gender) in collective teacher efficacy scales to handle bullying, in order to make meaningful comparisons across these school stakeholders.

### 1.4. External Validity

School climate is strongly related to bullying rates [54,55]. Scholars posit that the quality of school climate explains differences in teachers’ perceptions of collective efficacy to obtain school goals [21,56]. Scholars suggest that teachers who experience strong relationships in their school tend to perceive their school staff as having higher capabilities to deal with bullying [56,57,58]. Principal support is another essential factor influencing teachers’ perceptions of their own capability to handle bullying. Studies suggest that when teachers perceived support from their principal, their perception of efficacy to deal with bullying increased [59,60]. Finally, studies exploring school factors related to bullying have consistently reported that higher teacher perceptions of their capability to cope with bullying as a school team has lower bullying reports [32,35,43]. In this context, we analyzed the relationship between the scale and these school variables to assess the external validity of the scale.

### 1.5. The Present Study

In order to advance the current understanding of the effects of collective efficacy on bullying, the authors have considered the four main gaps found in the literature to conduct the study [24,25]. First, collective efficacy must measure teachers’ perceptions of staff capabilities rather than their ‘own’ competencies. Second, it is critical to avoid using teachers’ perceptions on locus of control or similar concepts instead of teachers’ perceptions about their group’s capability. Third, it is relevant to underline that the perception of teachers on collective efficacy is a specific construct [21,41] that has been evaluated in the context of bullying using scales that have not been developed for this purpose [43]. Consequently, it is impossible to know to what extent teachers’ perceptions effectively refer to collective efficacy in handling bullying while carrying out other teaching-related activities [17,27,42]. Four, to our knowledge, no scale measures teachers’ perception of staff collective efficacy to handle bullying. Considering all the above, it remains crucial to use an instrument that effectively measures teachers’ perceptions of collective efficacy to handle bullying, as teachers represent a unique population capable of accurately rating teaching staff abilities.

Thus, to close the gaps detected in previous studies [17,27,42] we developed a psychometrically sound scale to measure teachers’ perceptions of collective efficacy to handle bullying. Unlike other scales, this one is capable of (1) assessing the perceptions of teachers on collective efficacy rather than assessing teachers’ actual abilities, and (2) effectively assessing collective efficacy of teaching staffs to handle bullying. It is essential to mention that this scale was specifically developed to be responded to by teachers rather than students or other school staff members.

**Figure 1:**
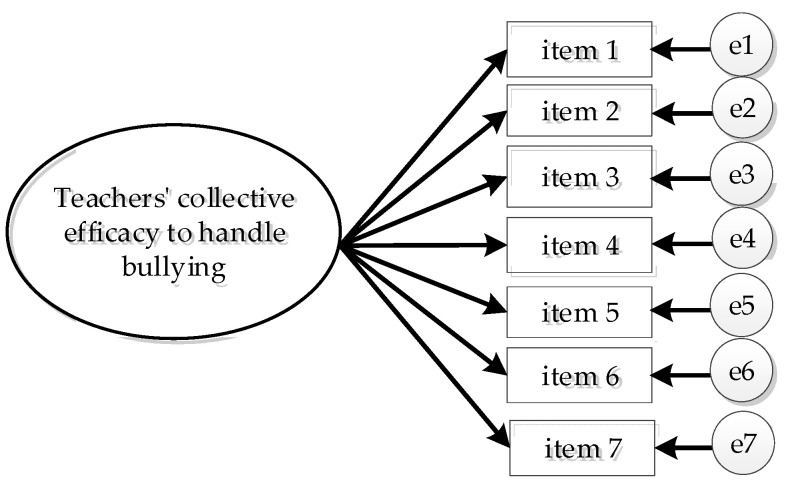
Factor Model of Teachers’ Perception of Collective Efficacy to Handle Bullying Depicting One First-Order Factor Model.

**Figure 2:**
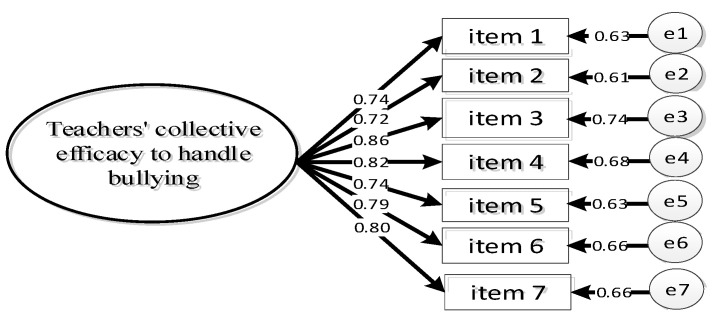
Factor Model of Teachers’ Perception of Collective Efficacy to Handle Bullying.

Psychometric properties of the scale were tested in the following ways: (1) the fit of the one-dimensional measurement model (see Figure 1); (2) cross-validation of the model in similar independent samples of teachers; (3) measurement invariance by gender; (4) latent means difference by gender in the perception of collective efficacy, when scalar invariance was confirmed; (5) discriminant and concurrent validity by examining the association of the construct with school climate, principal support, and bullying.

Five hypotheses were proposed based on the literature review. Hypotheses 1 (dimensionality): A one-dimensional first-order model has adequate goodness-of-fit to the data. Hypothesis 2 (cross-validation): The measurement is replicable on an independent teacher sample. Hypothesis 3 (measurement invariance): The measurement model is invariant across gender. Hypothesis 4 (latent means differences): Given the inconsistencies of previous studies, no previous hypothesis was made about gender differences. Hypothesis 5 (discriminant validity and concurrent validity): Results from the relationships with principal support, school climate, and bullying will allow for assuming discriminant and concurrent validity.

## 2. Materials and Methods

### 2.1. Participants

Teachers came from urban public elementary schools (*n* = 110) in four cities of Sonora, Mexico. The schools included low-Socio Economic Status (SES) and middle-SES students (National Institute for the Evaluation of Education, 2018). The study sample was randomly divided in two subsamples. The first sample (*n* = 402) was used for calibration purposes and comprised 190 (47%) males and 212 (53%) females, who aged from 25 to 73 years old (*M* = 37.9 years, *SD* = 11.2). The distribution by grade was as follows: 1st = 15%; 2nd = 16%; 3rd = 16%; 4th = 19%; 5th = 16%; 6th = 18%. The second sample (*n* = 402) was used for cross-validation analysis, which was comprised of 178 (44.3%) males and 224 (56%) females, aged from 25 to 76 years old (*M* = 36.4 years, *SD* = 9.1). The distribution by grade was as follows: 1st = 17%; 2nd = 16%; 3rd = 17%; 4th = 17%; 5th = 18%; 6th = 15%.

### 2.2. Measures

#### 2.2.1. Teachers’ Perception of Collective Efficacy Scale to Handle Bullying (TCEB)

Drawing on the work conducted by previous scholars [17,21], we developed a self-reported scale to measure teachers’ perceptions of collective efficacy to handle bullying (TCEB; see Table 1). The TCEB included 7 items that previously went through an expert judgment evaluation process to ensure the suitability and relevance of each item. The items showed a content validity index greater than 0.80 [61]. Through the scale, teachers were asked to share their perceptions of their teaching staff’s capabilities to carry out specific actions to handle bullying incidents inside the school (e.g., I believe that teachers in my school are capable of professionally facing bullying situations). Participants responded using a Likert-type scale (1 = Totally not capable to 5 = Completely capable).

#### 2.2.2. School Climate

Nine items were adapted from the California Staff Survey (CSS) for measuring school climate [62]. The collaborative and iterative technique was used to translate the scale from English to Spanish [63]. The items were grouped into three dimensions: (a) Cohesion and support, respect, and willingness to help other school members, (3 items, e.g., When students have a problem, they feel confident asking teachers for help; McDonald Omega coefficient (ω) = 0.76; average variance extracted (AVE) = 0.64), (b) Structure, equity and justice in disciplinary norms and practice (3 items, e.g., Students at this school were admonished when they violated school rules; McDonald’ Omega ω = 0.74; average variance extracted AVE = 0.55), and (c) Academic engagement, students’ engagement with the school (3 items, e.g., Students like school, ω = 0.71, AVE = 0.51). Participants responded using a Likert scale with five options (1 = Totally disagree to 5 = Totally agree). Goodness-of-fit statistics supported the scale adjustment to the data, Satorra–Bentler statistic (SBX^2^). = 37.39, *df* = 16, *p* = 0.002; comparative fit index (CFI) = 0.98; standardized root mean square residual (SRMR) = 0.018; Tucker–Lewis index (TLI) = 0.97; root mean square error of approximation (RMSEA) = 0.045, 90% CI [0.025, 0.065]).

#### 2.2.3. Principal’s Support

Principal’s Support Scale (PSS) [64], adapted according to [65], was used to measure teachers’ perceptions of the support from principals in anti-bullying activities. The scale comprises 7 items (e.g., “Principal gives me feedback to improve my skills to handle bullying”; ω = 0.90, AVE = 0.53) in a Likert-type response format (1 = never to 5 = always). The confirmatory factorial analysis showed that one-dimensional latent model represents an adequate measure of the construct (SBX^2^ = 15.90, *df* = 11, *p* = 0.145; CFI = 0.99; SRMR = 0.014; TLI = 0.98; RMSEA = 0.049, 90% CI [0.025, 0.055]).

#### 2.2.4. Bullying

The Teacher Bullying Report Scale [66] was used. The scale comprises indicators of direct (4 items, e.g., Students in my school damage other students’ belongings) and indirect (4 items, e.g., Students in my school spread negative rumors about other students) bullying situations. Teachers responded to how frequently these situations occurred in their school. Participants responded using a five-point Likert scale (1 = Never to 5 = Always). The collaborative and iterative technique was used to translate the scale from English to Spanish [62]. The CFA confirmed an acceptable adjustment of the latent model to the data (SBX^2^ = 19.23, *df* = 8, *p* = 0.014; SRMR = 0.016; CFI = 0.99; TLI = 0.98; RMSEA = 0.05, 90% CI [0.026, 0.071]). The scale reliability was also acceptable (ω = 0.83, AVE = 0.58).

### 2.3. Procedures

The study gained ethical approval from the Ethical Committee of the Technological Institute of Sonora (Number 2020_0014). Elementary teachers from 110 schools were recruited by email to participate in this study. Teachers who accepted participation signed a consent letter and then responded to the questionnaires online through Qualtrics software. Personal identifiers were removed to protect participants’ confidentiality. The teachers’ sample was randomly divided into two subsamples. We used a subsample for calibration purposes (internal and external validity analysis) and the other one for cross-validation (examining model replicability).

### 2.4. Data Analysis

Patterns of missing data were (less than 5% in all variables) verified to be entirely at random; then the multiple imputation method, available in SPSS 26, was used to analyze data. To estimate goodness-of-fit confirmatory factorial analysis (CFA), a robust maximum likelihood estimator (MLR) was performed in Mplus 8. Mplus software was also used to estimate the non-biased standards error in the nested data. The goodness of model fit was tested using *X*^2^ based on the Satorra–Bentler statistic (SBX^2^). Also, we assessed the model fit using goodness-of-fit indices, including: the comparative fit index (CFI ≥ 0.95), the Tucker–Lewis index (TLI ≥ 0.90), standardized root mean square residual (SMRM ≥ 0.08), and root mean square error of approximation (RMSEA ≤ 0.08) [67,68]. Furthermore, we tested the McDonald Omega coefficient (ω ≥ 0.70) and average variance extracted (AVE ≥ 0.50), which together ensure adequate reliability of the measure [69,70].

A multigroup test strategy was used to assess the replicability of measurement models across samples. We also compared two independent teacher testing samples, the unconstrained model with constrained models (factor loadings and variances/covariances fixed). Factorial invariance implies that SBX^2^ did not differ significantly (*p* > 0.001), ΔCFI ≤ 0.01, and ΔRMSEA ≤ 0.015 [71].

#### 2.4.1. Measurement Invariance by Gender

We examined measurement invariance by gradually comparing a restrictive model. Based on the SEM literature [71], we examined configurational invariance to prove that the same number of factors and variables are similar across groups (configurational invariance). If the baseline model fit the data, we compared their adjustment when a factor loading was fixed across male and female groups (metric invariance). After verifying metric invariance, we evaluated a model with constraints in the intercept (scalar invariance). These models were compared using ΔSBX^2^. Differences in the SBX^2^ were not statistically significant (ΔSBX^2^ with *p* > 0.001), suggesting that constraints imposed were equivalent across the group [68,71]. However, the ΔSBX^2^ is sensitive to larger samples; scholars have advocated the use of goodness-of-fit, such as differences in CFI (ΔCFI < 0.01) and differences in RMSEA (ΔRMSEA < 0.015) [69,71]. Finally, if scalar invariance was confirming, we calculated latent means differences by gender. The scalar invariance implies that differences in the means are explained by measurement factors [72,73].

#### 2.4.2. Discriminant Validity Analysis

As suggested in the literature [70], we examined the discriminant validity of TCEB with similar constructs such as principal support and school climate. We assumed that the discriminant validity of the scale relies on the square of correlation being less than the average extracted (AVE) from the other scales.

#### 2.4.3. Concurrent Validity

The evidence of concurrent validity concerns the association of the scale in an expected way with other similar and different constructs [74]. Thus, we analyzed the TPCE with principal support, school climate, and bullying. We evaluated the effects based on the cut-off rules proposed [75], which suggest that an *r* of 0.10 indicates a small effect size, *r* = 0.30 a medium effect size, and *r* of 0.50 a large effect size.

## 3. Results

### 3.1. Descriptive Analysis

Descriptive statistics indicate that teachers perceived their teaching staff capable of handling bullying events (see Table 2). Based on values of distribution (skewness and kurtosis and standard error), we calculated *z* value, which resulted in no significance in any items (*p* > 0.001), indicating a univariate normal distribution (Hair et al., 2010; Ho, 2014).

### 3.2. Dimensionality Analysis

We calculated a confirmatory factor analysis with the calibration sample (*n* = 402). The fit of a one-dimensional first-order measurement model was verified (SBX^2^ = 26.35, *df* = 12, *p* = 0.010; SMRM = 0.019; CFI = 0.99; TLI = 0.98; RMSEA = 0.048, 90% CI [0.026, 0.069]). The factor loadings ranged from 0.72 to 0.86, which were significant (*p* < 0.001) (see Figure 2). Reliability of the scale was acceptable (ω = 0.80, AVE = 0.61).

### 3.3. Cross-Validation Analysis

We used a multigroup approach to examine the model’s internal structure replicability in an independent sample of teachers. Multigroup analysis results offered evidence of configurational (SBX^2^ = 35.09, *df* = 22, *p* = 0.038; SMRM = 0.021; CFI = 0.99; TLI = 0.98; RMSEA = 0.038, 90% CI [0.009, 0.061]), metric and scalar invariance (see Table 3). These findings confirm that the factorial model is replicable in cross-validation samples.

### 3.4. Measurement Invariance by Gender

Multigroup analysis results showed that the factor model is invariant in both genders (see Table 4). The acceptable goodness-of-fit statistics of the baseline model (configurational invariance), suggests a similar factorial structure by gender (SBX^2^ = 45.85, *df* = 24, *p* = 0.008; SRMR = 0.024; CFI = 0.98; TLI = 0.97; RMSEA = 0.043, 90% CI [0.032, 0.067]). Then, factor loadings were fixed to be equal across gender (metric invariance), the ΔSBX^2^ between configurational and metric models were not statistically significant (*p* > 0.001), and the differences of the CFI and RMSEA values were smaller than 0.01 and 0.015, respectively, suggesting the existence of metric invariance. Finally, we constrained the intercept across groups (scalar invariance), the ΔSBX^2^ between the metric model and scalar was not statistically significant (*p* > 0.001); there were also no critical changes in CFA and RMSEA.

### 3.5. Means Latent Differences

The means of the male group were fixed to zero, while the mean of the female group was estimated freely. The latent means of the female teachers informed differences in values. The results showed no statistically significant differences by teachers’ gender on the measurement model (teachers’ perception of collective efficacy to handle bullying = 0.17, *z* = 1.09, *p* = 0.276, *d* de Cohen = 0.11).

### 3.6. Discriminant and Concurrent Validity

Table 5 shows that *R*^2^ between the scales was lower than AVE, indicating evidence of discriminant validity according to the standard rules proposed in the literature (see Hair et al., 2010). These results suggest that TCEB measures a specific whole-school factor construct. Regarding concurrent validity, we found that the teachers’ perceptions of collective efficacy to handle bullying were positively associated with principal support and school climate, and negatively with bullying rates. According to Cohen (1998), results indicate that the effect size of these correlations ranged between small (*r* > 0.10) and medium (*r* > 0.30), which has explicative and applied consequences.

## 4. Discussion

This study assesses the validity and reliability evidence of teachers’ perceptions of collective efficacy scale to handle bullying (TPCB). Findings confirmed the goodness-of-fit to a one-dimensional first-order model. Furthermore, results show this model functions similarly for both genders and confirmed no significant differences in the means between female and male teachers. Finally, the scale relations with other relevant whole-school constructs and bullying provided discriminant and concurrent validity evidence.

### 4.1. Dimensionality Hypothesis

As suggested [19], collective efficacy refers to group members’ beliefs about their capability to achieve certain tasks and must be measured by particular domain functioning. Thus, we expected that a one-dimensional model would be helpful to measure teachers’ perceptions of collective efficacy to handle bullying. The items of the scale focused on evaluating the teachers’ perceptions of their efficacy as a group to carry out different actions to cope with bullying. CFA results supported the unidimensional structure of the scale. The validation findings supported the dimensionality on two independent samples of teachers. These results confirmed cross-sample replicability of the factorial model, indicating internal validity of the scale [70].

However, our findings are contradictory to reports of scales based on the theory [44], which divided the construct into two dimensions (cohesion and social control) [35,38,44]. Similar to recent research [76], we think these scales evaluate the group’s conditions or characteristics that allow developing collective teacher efficacy, but not the perception of group capability. Even so, further studies should integrate the two perspectives to better understand the construct.

### 4.2. Model Invariance by Gender

Our findings provide empirical evidence supporting the invariance by gender of the TCEB. Thus, these findings indicate the scale is reliable to make accurate comparisons between male and female teachers. Although prior research on collective teacher efficacy by gender has been inconsistent [48,50], our results are in line with previous research [77,78,79] that suggested gender has not influenced teachers’ group efficacy in handling bullying. We assume these contradictory results are expected because most previous research did not report scale invariance by gender. Therefore, their result might not reflect actual group differences regarding the construct. However, further studies are necessary to explore teachers´ perceptions of their collective efficacy in diverse cultural and educational settings using invariant measurement scales.

### 4.3. Discriminant Validity

The relationships between the TCEB scale and the school variables (principal support and school climate) showed discriminant validity of the scale. These findings suggest that teachers’ perceptions of collective efficacy to handle bullying is a uniqueness school construct. Future bullying research should analyze the variables that encourage or hinder the perceptions of teacher collective efficacy to handle bullying, and their influence on other school constructs involved in bullying.

### 4.4. Concurrent Validity

The positive correlation of TCEB with school climate and principal support, and its negative correlation with bullying rates provide evidence of concurrent validity of the scale. Overall, our findings align with previous research [34,43,57,58,59], which suggests that teachers’ collective efficacy to handle bullying impacts school functioning and student bullying behavior. Specifically, our findings indicate that the quality of school climate influences teachers’ collective efficacy. We believe that when teachers perceive school relationships as caring and respectful, this condition improves their sense of engagement with the school community. As a result, teachers tend to perceive school staff members as more qualified to cope with bullying events [56,57,58]. Indeed, teachers’ collective beliefs on staff efficacy in assisting victims and discouraging aggressive behaviors resulted in a critical factor in explaining difference rates in school bullying. Another important finding is that results confirm the prominent role of the principal in bullying prevention. Our study suggests that the support that principals offer to teachers in antibullying interventions has a positive effect on teachers’ collective efficacy to handle bullying [59,60,65]. These results evinced that teachers’ perception of collective efficacy to handle bullying is a whole-school construct associated with theoretical and practical consequences for school dynamics and student behavior.

### 4.5. Theoretical and Practical Implications

In line with SCT [18,19], results extend from analyzing factors associated with the human agency to collective agency. The study confirms that teachers’ shared beliefs about teachers’ staff capability to achieve collective goals are essential for the collective agency. Moreover, the study suggests that the collective efficacy construct is helpful to explain teachers’ and students’ interpersonal behavior. Additionally, the study shows the values of the development task-specific measures of collective teacher efficacy.

From a practical point of view, this study provides psychometric ground measure of teachers’ perceptions of collective efficacy to handle bullying. This measure is essential for identifying other variables that can explain it; further research should deepen understanding of school factors that can promote it and its consequences on students’ behavior and school functioning. Teachers’ collective beliefs in staff members’ efficacy in assisting the victims and discouraging aggressive behaviors are associated with a positive whole-school variable and less aggressive behavior. Although further research is necessary, our results suggest that teachers’ collective efficacy hinders bullying directly and indirectly by their association with a positive school climate.

### 4.6. Limitations

Despite its contributions to the study of collective efficacy and bullying, this research has some limitations. First, the scale is based on self-reported data. Therefore, results may be affected by social desirability. It is advisable to compare the scale findings with measures of the construct based on the perceptions of other school members (e.g., students and principals). Second, the sample is comprised of only teachers from urban schools in Mexico. In this regard, it is critical to cross-validate the scale in samples of teachers from different contexts, such as rural and indigenous communities of Mexico.

Researchers have underlined the value of examining cultural differences on teacher efficacy measurements [80,81]. Thus, future research should examine the scale’s psychometric properties across samples of different countries [82]; this would allow researchers to determine if there are cultural discrepancies in teacher perceptions and handling bullying.

In addition, it will be helpful to add more items related with different types of bullying (e.g., physical, social, and psychological) [40] or the nature of the task (e.g., identify, prevent or intervene in bullying events) [78], to identify if there are differences in perceptions of the TCEB depending on these factors. Finally, further studies are necessary to standardize the scale for practical use.

## 5. Conclusions

Previous studies suggest that collective teacher efficacy is an essential whole-school factor related to bullying [31,32,33,34,35,36,38,39,40,43]. However, this construct has been scarcely explored in bullying literature [31]. The study provides a theoretical and robust instrument that may allow scholars to deepen the understanding of school factors that can reduce bullying. Thus, using this instrument may generate knowledge that can contribute to developing a whole-school intervention program aimed to reduce bullying events. Finally, given the growing evidence on the effectiveness of collective efficacy in preventing bullying events, we encourage other scholars to assess collective teacher efficacy in future research when assessing the effectiveness of the antibullying intervention.

## Figures and Tables

**Table 1:** Teachers’ Perception of Collective Efficacy to Handle Bullying Scale.

Item	Totally Not Capable	Not Capable	More or Less Capable	Capable	Totally Capable
Instruction:					
How much do you perceive you and the teaching staff of your school are capable to realize the following actions?					
In a bullying situation, stop even the most challenging student.					
Prevent incidents of bullying.					
Manage all types of bullying.					
Solve bullying situations effectively.					
Effectively apply the rules and procedures for handling with bullying.					
Effectively address the behavioral needs of students involved in bullying situations.					
Create an environment free of bullying, even in the most challenging groups.					

**Table 2:** Means, Standard Deviation, Skewness, and Kurtosis for Calibration Sample (*n* = 402).

Item	*M*	*SD*	Skewness	Kurtosis
Item 1	3.75	0.88	−0.52 (0.12)	0.16 (0.24)
Item 2	4.19	0.79	−1.11 (0.12)	2.00 (0.24)
Item 3	4.07	0.73	−0.68 (0.12)	1.12 (0.24)
Item 4	4.16	0.76	−0.74 (0.12)	0.68 (0.24)
Item 5	4.14	0.84	−0.91 (0.12)	0.67 (0.24)
Item 6	4.07	0.87	−1.08 (0.12)	1.33 (0.24)
Item 7	4.03	0.86	−0.96 (0.12)	1.22 (0.24)

**Table 3:** Goodness-of- Statistic for Testing Model Invariance Across Calibration Sample (*n* = 402) and Validation Sample (*n* = 402).

Model	SBX^2^	*df*	ΔSB*X*^2^	Δ*df*	*p*	ΔCFI	ΔRMSEA
Configurational	35.09	22			0.038		
Metric	37.83	29	2.74	7	0.20	0.005	0.012
Scalar	42.15	38	4.32	9	0.61	0.004	0.014

**Table 4:** Goodness-of-Statistic for Testing Measurement Invariance by Gender.

Model	SBX^2^	*df*	ΔSBX^2^	Δ*df*	*p*	ΔCFI	ΔRMSEA
Configurational	45.85	24			0.038		
Metric	51.71	31	9.86	7	0.341	0.005	0.012
Scalar	72.14	40	20.43	9	0.015	0.004	0.014

**Table 5:** Correlations Between Scales, Square Correlations, and Average Variance Extracted.

Variable	TCEB AVE = 0.54
TCEB	-
Principal support	0.29 *** (0.08)
School climate	0.40 *** (0.25)
Bullying	−0.24 *** (0.06)

Note: TCEB = Teachers’ perception of collective efficacy to handle bullying. Squared correlations (*R*^2^) are reported in parentheses. *** *p* < 0.001.

## Data Availability

The data presented in this study are available on request from the corresponding author. The data are not publicly available due to the funder demand.
